# Neurometabolite changes in response to antidepressant medication: A systematic review of ^1^H-MRS findings

**DOI:** 10.1016/j.nicl.2023.103517

**Published:** 2023-09-25

**Authors:** Daphne E. Boucherie, Liesbeth Reneman, Henricus G. Ruhé, Anouk Schrantee

**Affiliations:** a Amsterdam UMC, Location AMC, Department of Radiology and Nuclear Medicine, Meibergdreef 9, 1109 AZ Amsterdam, the Netherlands; b Department of Psychiatry, Radboudumc, Radboud University, Reinier Postlaan 4, 6525 GC Nijmegen, the Netherlands; c Donders Institute for Brain Cognition and Behaviour, Radboud University, Kapittelweg 29, 6525 EN Nijmegen, the Netherlands

**Keywords:** Major Depressive Disorder, SSRI, SNRI, (es)ketamine, ^1^H-MRS, Glutamate, GABA

## Abstract

Selective serotonin reuptake inhibitors (SSRIs), serotonin and noradrenaline reuptake inhibitors (SNRIs), and (es)ketamine are used to treat major depressive disorder (MDD). These different types of medication may involve common neural pathways related to glutamatergic and GABAergic neurotransmitter systems, both of which have been implicated in MDD pathology. We conducted a systematic review of pharmacological proton Magnetic Resonance Spectroscopy (^1^H-MRS) studies in healthy volunteers and individuals with MDD to explore the potential impact of these medications on glutamatergic and GABAergic systems. We searched PubMed, Web of Science and Embase and included randomized controlled trials or cohort studies, which assessed the effects of SSRIs, SNRIs, or (es)ketamine on glutamate, glutamine, Glx or GABA using single-voxel ^1^H-MRS or Magnetic Resonance Spectroscopic Imaging (MRSI). Additionally, studies were included when they used a field strength > 1.5 T, and when a comparison of metabolite levels between antidepressant treatment and placebo or baseline with post-medication metabolite levels was done. We excluded animal studies, duplicate publications, or articles with ^1^H-MRS data already described in another included article. Twenty-nine studies were included in this review. Fifteen studies investigated the effect of administration or treatment with SSRIs or SNRIs, and fourteen studies investigated the effect of (es)ketamine on glutamatergic and GABAergic metabolite levels. Studies on SSRIs and SNRIs were highly variable, generally underpowered, and yielded no consistent findings across brain regions or specific populations. Although studies on (es)ketamine were also highly variable, some demonstrated an increase in glutamate levels in the anterior cingulate cortex in a time-dependent manner after administration. Our findings highlight the need for standardized study and acquisition protocols. Additionally, measuring metabolites dynamically over time or combining ^1^H-MRS with whole brain functional imaging techniques could provide valuable insights into the effects of these medications on glutamate and GABAergic neurometabolism.

## Highlights

• SSRIs and SNRIs do not consistently affect Glu/GABA systems in ^1^H-MRS studies.
• Amidst null findings, we find some evidence that (es)ketamine increases ACC Glu.
• Standardization of study-, and acquisition protocols for ^1^H-MRS is essential.

## 1 Introduction

Major depressive disorder (MDD) is a debilitating and highly prevalent psychiatric disorder. Selective serotonin reuptake inhibitors (SSRIs) and serotonin and noradrenaline reuptake inhibitors (SNRIs) are first-line classes of antidepressants, commonly prescribed for the treatment of MDD and other mood disorders. Although the mechanism of action of SSRIs is not fully understood, they selectively block the reuptake of serotonin from the synaptic cleft, whereas SNRIs additionally inhibit the noradrenaline transporter. This results in increased levels of serotonin (and noradrenaline) in the synaptic cleft, which is thought to be associated with their antidepressant properties. While SSRIs and SNRIs are generally considered safe and effective, not all patients respond to treatment with these types of medication (Al-Harbi, 2012).

(Es)ketamine is another type of antidepressant medication that has gained attention in recent years due to its rapid onset of action and effectiveness in treatment-resistant depression (TRD) (Berman et al., 2000, Zarate et al., 2006). (Es)ketamine is a dissociative anesthetic that works by non-selectively antagonizing the N-methyl-D-aspartate (NMDA) receptor, a glutamatergic receptor in the brain (Orser et al., 1997). These receptors are predominantly situated on GABAergic parvalbumin interneurons (Grunze et al., 1996, Ng et al., 2018). The NMDA antagonism results in increased glutamate release, which is thought to enhance synaptic plasticity and neuroplasticity (Buck et al., 2006, Keilhoff et al., 2004) and has been implicated in (es)ketamine’s antidepressant effects. Although the exact mechanism of action of (es)ketamine is not fully understood either, its rapid onset of action and effectiveness in treating TRD have made it a promising avenue for the development of new antidepressant medication.

Alterations in glutamatergic and GABAergic systems, through downstream or direct engagement, have been proposed as a potential overlapping mechanism of SSRIs, SNRIs and (es)ketamine antidepressant medications (Skolnick et al., 1996). Indeed, results from animal, post-mortem, imaging, pharmacological, and genetic studies linking alterations in glutamatergic and GABAergic systems to the pathology of MDD have led to the glutamate hypothesis of depression, which suggests an altered glutamatergic metabolism as a mediator of MDD pathology (Sanacora et al., 2012). Interestingly, proton Magnetic Resonance Spectroscopy (^1^H-MRS), a non-invasive neuroimaging technique that enables direct measurements of metabolite levels *in vivo*, has provided evidence of altered glutamatergic metabolites and lower GABA levels in patients with MDD (Lener et al., 2017, Moriguchi et al., 2019, Ritter et al., 2022, Yüksel and Öngür, 2010). However, a systematic review of studies about the effects of antidepressant medications on the glutamatergic and GABAergic systems has not been conducted so far.

In this systematic review, we investigate changes in glutamatergic and GABAergic neurotransmission induced by three types of antidepressant medications: SSRIs, SNRIs, and (es)ketamine. For this purpose, we collected and analyzed human (pharmacological) ^1^H-MRS findings to summarize all available evidence on changes in glutamate, glutamine, glutamate + glutamine (Glx), and GABA in response to acute administration of, or treatment with these medications. Our analysis aims to determine if alterations in these neurotransmitter systems represent a shared biological pathway between different types of antidepressant medication and their response in MDD. Firstly, we evaluate studies that assess the effect of antidepressants on metabolite concentrations in healthy volunteers and individuals with MDD. Secondly, we investigate the relation between metabolite concentrations and clinical outcomes.

## 2 Methods

This systematic review was conducted in accordance with the Preferred Reporting Items of Systematic Reviews and Meta-analyses (PRISMA) reporting guideline (Moher et al., 2009). The review protocol was registered in PROSPERO (CRD42022384696).

### 2.1 Search strategy

PubMed, Web of Science and Embase were searched from inception to January 10th 2023. See Table S1 (Supplementary Methods) for the full search terms and results per database. All titles and abstracts of retrieved publications were independently screened by two researchers (DB and AS) to assess eligibility for inclusion in Rayyan (Ouzzani et al., 2016). In case of inconsistencies, full-text articles were obtained. Full text articles were independently screened by AS and DB based on inclusion criteria below. Furthermore, reference lists of selected articles were screened for additional studies.

### 2.2 Eligibility criteria

Studies were included when 1) the study design was a randomized controlled trial or a longitudinal cohort study with pre- and post-measurements, 2) the study assessed the effects of SSRIs, SNRIs, or (es)ketamine on neurometabolism, 3) single-voxel ^1^H-MRS or Magnetic Resonance Spectroscopic Imaging (MRSI) was utilized to assess metabolite levels, 4) healthy volunteers or individuals primarily diagnosed with MDD were studied (studies including individuals with MDD with comorbid psychiatric disorders were not excluded). Additionally, studies were included when 5) glutamate, glutamine, Glx or GABA were amongst the investigated metabolites, 6) studies used a field strength > 1.5 T, and 7) when the analysis consisted of comparison of metabolite levels between antidepressant treatment and placebo or when comparing baseline with post-medication metabolite levels. We excluded animal studies, duplicate publications, or articles with ^1^H-MRS data already described in another included article.

### 2.3 Data extraction

Data extraction was done independently by DB and AS. We identified demographic and clinical characteristics: the number of participants, healthy volunteers versus individuals with MDD, medication status, medication administration (i.e., medication type, route of administration, dose and duration), experimental design, ^1^H-MRS parameters (e.g., field strength, sequence and voxel size), voxel placement, metabolites and quantification method (reference metabolite and software), and symptom severity measures. For the articles investigating the effect of SSRI and SNRI administration or treatment, the duration was classified as acute (single dose), subchronic (≤ 4 weeks), or chronic (> 4 weeks). Results were described for healthy volunteers and individuals with MDD separately.

### 2.4 Quality assessment of included studies

The NIH quality assessment tools *for controlled intervention studies* and *for before-after studies with no control group* were used (https://www.nhlbi.nih.gov/health-topics/study-quality-assessment-tools). These measures can be used to assess the risk of bias for each of the included studies. We additionally assessed the quality of MRS-reporting using the MRS-Q assessment tool (Peek et al., 2020, available at https://osf.io/8s7j9), a new quality appraisal tool based on consensus papers and expert opinion on best-practice. Studies were quantified as poor, fair, or good based on the NIH quality assessment tools.

## 3 Results

**Fig. 1:**
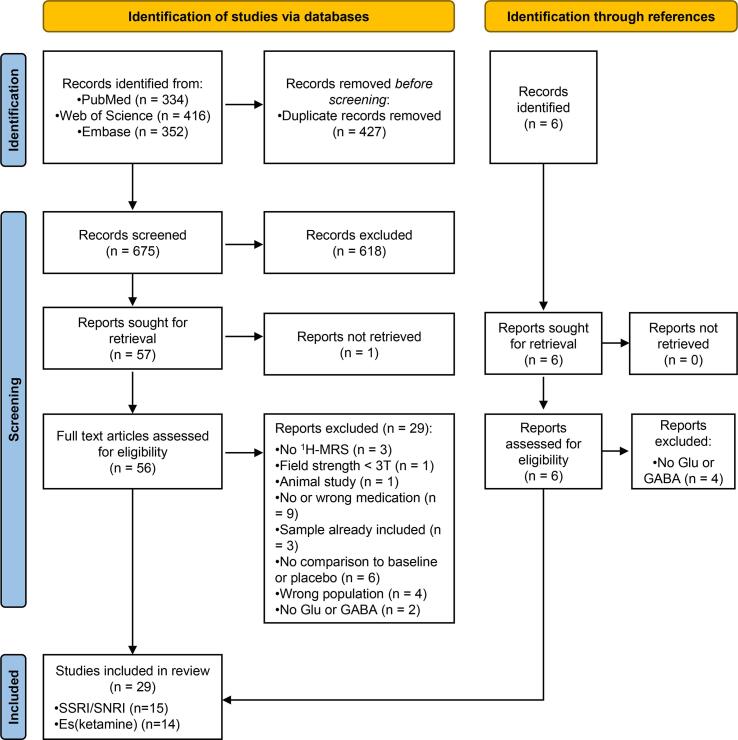
Literature PRISMA flowchart.

**Fig. 2:**
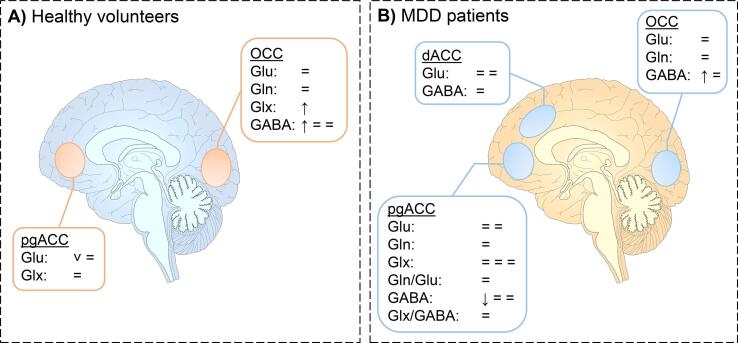
Changes in glutamate, glutamine, Glx and GABA following SSRI or SNRI administration or treatment in a) healthy volunteers and b) participants with MDD. Changes are depicted by symbols, with each symbol illustrating one finding. ↑illustrates a significant increase in metabolite concentration;^illustrates a trend increase (0.05 < p < 0.10); ↓ illustrates a significant decrease in metabolite concentration; v illustrates a trend decrease (0.05 < p < 0.10); = illustrates no changes in metabolite concentrations. dACC: dorsal anterior cingulate cortex; pgACC: pregenual anterior cingulate cortex; OCC: occipital cortex.

**Fig. 3:**
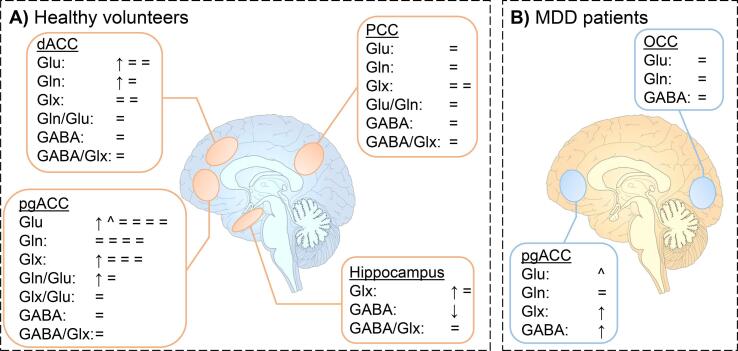
Changes in glutamate, glutamine, Glx and GABA following (es)ketamine administration in A) healthy volunteers and B) participants with MDD. Changes are depicted by symbols, with each symbol illustrating one finding. ↑ illustrates a significant increase in metabolite concentration;^illustrates a trend increase (0.05 < p < 0.10); ↓ illustrates a significant decrease in metabolite concentration; v illustrates a trend decrease (0.05 < p < 0.10); = illustrates no changes in metabolite concentrations. dACC: dorsal anterior cingulate cortex; pgACC: pregenual anterior cingulate cortex; PCC: posterior cingulate cortex; OCC: occipital cortex.

The PRISMA flowchart (Fig. 1) summarizes study selection (Moher et al., 2009). Twenty-seven studies met the above-mentioned criteria. Additionally, 2 articles were included after screening the references lists of included articles, yielding a total of 29 included articles. Extracted features are shown in Table 1, Table 2 and findings are visualized in Fig. 2, Fig. 3. Unless otherwise specified, the studies that we report were conducted at 3 T.

**Table 1:** Outcomes of included studies investigating the effect of SSRI or SNRI administration or treatment on glutamatergic or GABAergic metabolites.

Article	Sample	Design	Medication	Dose	Duration	^1^H-MRS timepoint	Field strength and ^1^H-MRS sequence	VOI and voxel size (mm)	Metabolites	Quantification method	Results
**HC**											

Bhagwagar et al. (2004)	HC N = 10	randomized, placebo- controlled, double-blind crossover design	*i.v.* citalopram	10 mg	30 min	40-65 min p.i.	3T DQF PRESS	OCC 30 × 30 × 30	GABA	MRUI VARPRO	↑ GABA after citalopram infusion
Hansen et al. (2016)	HC N = 20	randomized, placebo- controlled, double-blind crossover design	Oral venlafaxine	75 mg/day	5 days	Post- treatment	3T PRESS	pgACC 20 × 20 × 20 Insula 15 × 20 × 50 PFC 15 × 25 × 20	Glu	LCModel	v Glu in pgACC, PFC and insula between placebo and venlafaxine
Maron et al. (2016)	HC N = 15	open-label pre-post design without placebo	Oral escitalopram	10 mg/day	7-10 days	Post- treatment	3T SPECIAL	OCC 20 × 25 × 20	Glu, Gln, GABA	LCModel	= Glu, Gln and GABA between baseline and post-treatment
Spurny et al. (2021)	HC N = 79	randomized, placebo- controlled, pre-post design	Oral escitalopram	10 mg/day	3 weeks	Post- treatment	3T MEGA-LASER MRSI	Hippocampus, insula, putamen, pallidum, thalamus 10 × 10 × 10	Glx, GABA+	LCModel	↓ GABA HIPP from baseline to post-treatment = GABA insula, putamen, pallidum and thalamus from baseline to post-treatment = Glx HIPP, insula, putamen, pallidum and thalamus from baseline to post-treatment
Taylor et al. (2008)	HC N = 30	randomized, placebo- controlled, parallel group design	Oral citalopram	20 mg/day	7-10 days	Post- treatment	3T PRESS 3T MEGA-PRESS	OCC n.s.	Glx, GABA	LCModel AMARES	↑ Glx in citalopram group compared to placebo and reboxetine groups after treatment = GABA post-treatment across all treatment groups
			Oral Reboxetine	8 mg/day							
Taylor et al. (2010)	HC N = 23 SSRI N = 10 PLAC	randomized, placebo- controlled, parallel group design	Oral citalopram	20 mg/day	7-10 days	Post- treatment	3T PRESS 3T J-PRESS	pgACC 20 × 20 × 20	Glu, Glx	LCModel AMARES	= Glx and GABA between baseline and post-treatment across groups

**MDD**											

Block et al. (2009)	MDD N = 5 SSRI N = 5 TCA	pre-post design without placebo	Oral citalopram	Mean 20 mg/day	8 weeks	Post- treatment	3T PRESS	HIPP 28 × 17 × 13	Gln, Glx	AMARES	= Gln and Glx between baseline and post-treatment No differences between groups No correlation between baseline Gln or Glx and baseline BDI No correlation between ΔGln or ΔGlx and ΔBDI No correlation between baseline Gln or Glx and ΔBDI
			Oral nortriptyline	Mean 105 mg/day							
Brennan et al. (2017)	MDD N = 16	open-label pre-post design without placebo	Oral Citalopram	20-40 mg/day	6 weeks	Day 3, 6, 42	3T J-PRESS 3T MEGA-PRESS	pgACC 20 × 20 × 20	Glu, Gln, GABA, Gln/Glu	LCModel	↓ GABA from baseline to day 3 of treatment = Glu, Gln, and Gln/Glu from baseline to day 3 of treatment = Glu, Gln, GABA, and Gln/Glu from baseline to day 6 and day 42 of treatment Association between ΔGABA (baseline - day 3) and ΔGABA (baseline - day 7) and ΔMADRS (baseline - day 42) Association between baseline GABA and ΔMADRS
Draganov et al. (2020)	MDD N = 18	open-label pre-post design without placebo	Variable, mostly oral escitalopram	Variable, mostly 15 mg/day	1 year	Post- treatment	3T PRESS	pgACC 20 × 20 × 20	Glu, Glx, GABA	TARQUIN	= Glu, Glx and GABA between baseline and post-treatment No correlation between ΔGlu, ΔGlx, or ΔGABA and ΔHDRS
Godlewska et al. (2015)	MDD N = 39	open-label pre-post design without placebo	Oral citalopram	10 mg/day	6 weeks	Post-treatment	3T SPECIAL	OCC 20 × 25 × 20	Glu, Gln, GABA	LCModel	= Glu, Gln, GABA between baseline and post-treatment No correlation between ΔGlu, ΔGln, or ΔGABA and ΔHDRS
Grimm et al. (2012)	MDD N = 14	open-label pre-post design without placebo	Oral SSRI (78%), SNRI (64%), TCA (28%), atypical neuroleptics (57%)	Variable	4 weeks	Post- treatment	3T PRESS	dACC 25 × 40 × 20 dlPFC 20 × 20 × 20	Glu	In-house time domain- frequency fitting	= dACC and dlPFC Glu from baseline to post-treatment No correlation between Glu and HDRS at baseline or post-treatment ↑ dlPFC Glu in responders vs non-responders post-treatment
Narayan et al. (2022)	MDD N = 39 SSRI N = 42 PLAC	randomized, placebo- controlled, double-blind parallel group design	Oral escitalopram	w1 10 mg/day w2-3 20 mg/day w4-8 30 mg/day	8 weeks	Post- treatment	3T MEGA-PRESS	pgACC 20 × 20 × 30	Glx, GABA, Glx/GABA	TARQUIN	= Glx, GABA, and Glx/GABA from baseline to post-treatment No correlation between ΔGlx or ΔGABA and ΔHDRS
Sanacora et al. (2002)	MDD N = 11	open-label pre-post design without placebo	Oral citalopram	Mean 24 mg/day	5 weeks	Post- treatment	2.1T J-edited ISIS	OCC 15 × 30 × 30	GABA	n.s.	↑ GABA from baseline to post-treatment No correlation between ΔGABA and ΔHDRS No correlation between baseline GABA and ΔHDRS
			Oral fluoxetine	Mean 27 mg/day							
Smith et al. (2021)	MDD N = 9	open-label pre-post design without placebo	Oral citalopram	w1 10 mg/day w2-12 20-40 mg/day	10-12 weeks	Post- treatment	7T STEAM	dACC 28 × 20 × 16 PCC 28 × 20 × 16	Glu, GABA	LCModel	= Glu and GABA in dACC and PCC between baseline and post-treatment Association between PCC ΔGlu and ΔBDI
Taylor et al. (2012a)	MDD N = 21 SSRI N = 19 PLAC	randomized, double-blind parallel group design	Oral escitalopram	10 mg/day	7 days	Post- treatment	3T PRESS	pgACC 30 × 30 × 20	Glx	LCModel	= Glx between baseline and post-treatment

Abbreviations: BDI: Beck Depression Inventory; dACC: dorsal anterior cingulate cortex; Glu: glutamate; Gln: glutamine; Glx: glutamate + glutamine; GABA: γ-aminobutyric acid; HC: healthy volunteers; HDRS: Hamilton Depression Rating Scale; *i.v.*: intravenous; MADRS: Montgomery-Asberg Depression Rating Scale; MDD: Major Depressive Disorder; n.s.: not specified; OCC: occipital cortex; PCC: posterior cingulate cortex; pgACC: pregenual anterior cingulate cortex; PFC: prefrontal cortex; p.i.: post-infusion; PLAC: placebo; POMS: Profile of Mood State; SNRI: serotonin and noradrenaline reuptake inhibitor; SSRI: selective serotonin reuptake inhibitor; TCA: tricyclic antidepressant; tCr: total creatine.

**Table 2:** Outcomes of included studies investigating the effect of (es-)ketamine administration on glutamatergic or GABAergic metabolites.

Article	Sample	Design	Medication	Dose	Duration	^1^H-MRS timepoint	Field strength and ^1^H-MRS sequence	VOI and voxel size (mm)	Metabolites	Quantification method	Results
**HC**											

Bednarik et al. (2021)	HC N = 12	open-label pre-post design without placebo	*i.v.* Racemic ketamine	0.8 mg/kg	50 min.	3 h post- infusion	3 T sLASER	PCC 22 × 22 × 22	Glu, Gln, Glx, Glu/Gln	LCModel	= Glu, Gln, Glx, and Glu/Gln from baseline to post-infusion
Bojesen et al. (2018)	HC N = 25	open-label pre-post design without placebo	*i.v.* Es-ketamine	0.25 mg/kg + 0.125 mg/kg	20 min + 20 min	During bolus 2x during infusion	3 T PRESS	pgACC 20 × 20 × 20	Glu, Gln, Glx	LCModel	= Glu, Gln, Glx between baseline and during bolus or during infusion
Colic et al. (2019)	HC N = 40 KET N = 40 PLAC	randomized, placebo- controlled, double-blind parallel group design	*i.v.* Racemic ketamine	0.5 mg/kg	40 min.	24 h post- infusion	7 T STEAM	pgACC 20 × 15 × 20	Glu	LCModel	↑ Glu in ketamine group compared to placebo group
Evans et al. (2018)	HC N = 17	randomized, placebo- controlled, double-blind crossover design	*i.v.* Racemic ketamine	0.5 mg/kg	n.s.	24 h post- infusion	7 T PRESS	pgACC 20 × 20 × 20	Glu, Gln	In-house linear combination program	= Glu from baseline to post-administration = Gln between baseline and post-administration
Gärtner et al. (2022)	HC N = 16	open-label pre-post design without placebo	*i.v.* Es-ketamine	0.12 mg/kg + 0.25 mg/kg/h	n.s. + d.s.	25 min. post start infusion	3 T J-PRESS	pgACC 25 × 18 × 20	Glu, Gln, Gln/Glu	Profit 2 (Prior knowledge Fitting)	^Glu from baseline to during infusion = Gln and Gln/Glu from baseline to during infusion
Javitt et al. (2018)	HC N = 31 KET N = 16 PLAC	randomized, placebo- controlled, parallel group design	*i.v.* Racemic ketamine	0.23 mg/kg + 0.58 mg/kg/h + 0.29 mg/kg/h	1 min. + 30 min. + 29 min.	4x during infusion	3 T PRESS	pgACC 25 × 30 × 25	Glx	LCModel	↑ Glx from baseline to first infusion measurement (0–15 min.) in ketamine group compared to placebo group = Glx from baseline to last 3 infusion measurements (15–60 min.) between groups = Glx from baseline to total infusion (60 min.) between groups
Kraguljac et al. (2017)	HC N = 15	placebo- controlled crossover design	*i.v.* Racemic ketamine	0.27 mg/kg + 0.25 mg/kg/h	10 min + d.s.	13 min. post start infusion	3 T PRESS	Hippocampus 26 × 15 × 10	Glx	AMARES	↑ Glx from baseline to during infusion
Li et al. (2017)	HC N = 12 KET N = 14 PLAC	randomized, placebo- controlled, double-blind parallel group design	*i.v.* Racemic ketamine	0.5 mg/kg	40 min.	1 h post- infusion 24 h post-in fusion	7 T STEAM	pgACC 20 × 15 × 10 dACC 25 × 15 × 10	Glu, Gln, Gln/Glu	LCModel	↑ pgACC Gln/Glu from baseline to 24 h post-infusion = pgACC Glu and Gln from baseline to 24 h post-infusion = pgACC and dACC Glu, Gln, and Gln/Glu from baseline to 1 h post-infusion = dACC Glu, Gln, and Gln/Glu from baseline to 24 h post-infusion
Rowland et al. (2005)	HC N = 10	placebo- controlled crossover design	*i.v.* n.s.	0.27 mg/kg + 0.135 mg/kg/h	20 min + d.s.	During bolus Start infusion	4 T STEAM	dACC 8 mL	Glu, Gln	n.s.	↑ Gln from baseline to during bolus = Glu from baseline to during bolus = Glu and Gln from baseline to start infusion
Silberbauer et al. (2020)	HC N = 25	pre-post design without placebo	*i.v.* Racemic ketamine	0.8 mg/kg	50 min.	2 h post- infusion	3 T MEGA-LASER MRSI	pgACC dACC PCC HIPP Thalamus Insula Putamen 160 × 260 × 160	Glx, GABA, GABA/Glx	LCModel	↓ HIPP GABA from baseline to post-infusion = HIPP Glx and GABA/Glx from baseline to post-infusion = pgACC, dACC, PCC, Thalamus, insula and putamen Glx, GABA and GABA/Glx
Stone et al. (2012)	HC N = 13	pre-post design without placebo	*i.v.* n.s.	0.26 mg/kg + 0.42 mg/kg/h	20 min + n.s.	25 min. post- infusion 35 min. post- infusion	3 T PRESS 3 T MEGA-PRESS	dACC 20 × 20 × 20 Thalamus 30 × 30 × 30	Glu, Gln, Glx, GABA	LCModel	↑ dACC Glu from baseline to post-infusion = dACC Glx from baseline to post-infusion = Thalamic GABA from baseline to post-infusion
Taylor et al. (2012b)	HC N = 8 KET N = 9 PLAC	randomized, placebo- controlled, parallel group design	*i.v.* n.s.	0.5 mg/kg	40 min.	3x during infusion	3 T PRESS 3 T J-PRESS	pgACC 30 × 20 × 20	Glu, Glx, Glx/Glu	LCModel AMARES	= Glu, Glx, and Glx/Glu between placebo and ketamine groups at any time point during infusion

**MDD**											

Evans et al. (2018)	MDD N = 20	randomized, placebo- controlled, double-blind crossover design	*i.v.* Racemic ketamine	0.5 mg/kg	n.s.	24 h	7 T PRESS	pgACC 20 × 20 × 20	Glu, Gln, Gln/Glu	In-house linear combination program	^Glu from baseline to post-administration = Gln between baseline and post-administration No relation between baseline Glu and infusion MADRS
Milak et al. (2016)	MDD N = 11	open-label pre-post design without placebo	*i.v.* Racemic ketamine	0.5 mg/kg	40 min.	3x during infusion post- infusion	3 T J-edited ISIS	pgACC 30 × 25 × 25	Glx, GABA	In-house pseudo- Voigt fit	↑ Glx from baseline to all timepoints during infusion ↑ GABA from baseline from 13 to 26 min. during infusion No correlation between ΔGlx or ΔGABA with ΔHDRS, ΔBDI or ΔPOMS
Valentine et al. 2011	MDD N = 10	placebo- controlled, single-blind crossover design	*i.v.* Racemic ketamine	0.5 mg/kg	40 min.	3 h post- infusion 48 h post- infusion	4 T J-edited ISIS	OCC 30 × 15 × 30	Glu, Gln, Gln/Glu, GABA	LCModel	= Glu, Gln, and GABA from baseline to all 3 h or 48 h post-infusion No correlation between ΔGlu, ΔGln, or ΔGABA with ΔHDRS No correlation between baseline Glu, Gln, or GABA and HDRS at 3 h or 48 h post-infusion

Abbreviations: BDI: Beck Depression Inventory; dACC: dorsal anterior cingulate cortex; d.s.: duration of scan; GABA: γ-aminobutyric acid; Glu: glutamate; Gln: glutamine; Glx: glutamate + glutamine; *i.v.*: intravenous; pgACC: pregenual anterior cingulate cortex; HC: healthy volunteers; HDRS: Hamilton Depression Rating Scale; KET: (es-)ketamine; MADRS: Montgomery-Asberg Depression Rating Scale; MDD: Major Depressive Disorder; n.s.: not specified; OCC: occipital cortex; PCC: posterior cingulate cortex; PFC: prefrontal cortex; PLAC: placebo; POMS: Profile of Mood States; VOI: volume of interest.

### 3.1 SSRIs and SNRIs

Fifteen of the included studies investigated the effect of SSRI or SNRI treatment on glutamatergic or GABAergic metabolite levels (Table 1, Fig. 2). Other metabolites are included in the Supplementary Results and Table S2.

#### 3.1.1 SSRIs and SNRIs in healthy volunteers

Six studies investigated the effect of SSRIs and SNRIs on glutamatergic or GABAergic metabolite levels in healthy volunteers, with most studies focusing on either the pregenual anterior cingulate cortex (pgACC) (2 studies) or the occipital cortex (OCC) (3 studies), which are therefore discussed separately (Table 1, Fig. 2a). All studies administered medication orally, except for one (Bhagwagar et al., 2004).

##### 3.1.1.1 Pregenual anterior cingulate cortex

In the first study, no effect of treatment on Glu or Glx in the pgACC was observed in participants receiving subchronic citalopram (SSRI; 20 mg/day; n = 23) or placebo (n = 10) for a duration of 7–10 days (Taylor et al., 2010). In contrast, a subchronic 5-day, randomized, placebo-controlled crossover study with venlafaxine (SNRI; 75 mg/day) observed a trend decrease in Glu in the pgACC compared to placebo in 20 participants (Hansen et al., 2016).

##### 3.1.1.2 Occipital cortex

In a study by Bhagwagar et al. (2004), acute intravenous citalopram (SSRI; 10 mg; n = 10) increased occipital GABA between 40 and 65 min after the citalopram infusion. In contrast, Taylor et al. (2008) found no treatment group differences in occipital GABA in 30 participants following 7–10 days of subchronic citalopram (SSRI; 20 mg/day) treatment. However, they observed an increase in Glx for the citalopram compared to the placebo group. Another study administering escitalopram (SSRI; 10 mg/day; n = 15) in a similar subchronic design, found changes in Glu, Gln and GABA concentrations from baseline to post-treatment (Maron et al., 2016).

##### 3.1.1.3 Other brain regions

Hansen et al. (2016) found a trend decrease in Glu in the prefrontal cortex (PFC) and insula after venlafaxine (SNRI; 75 mg/day) treatment compared to after placebo. Finally, Spurny et al. (2021) investigated Glx and GABA in 79 participants after three weeks of treatment with escitalopram (SSRI; 10 mg/day) using MRSI in five regions (insula, putamen, pallidum, thalamus and hippocampus), during an associative relearning paradigm. They observed a reduction in hippocampal Glx from pre- to post-treatment with escitalopram, but no effect on GABA. In the putamen, pallidum, and thalamus no differences between baseline and post-treatment were observed in Glx or GABA.

#### 3.1.2 SSRIs and SNRIs in individuals with MDD

Nine studies assessed the effect of SSRI or SNRI treatment on glutamatergic or GABAergic metabolites in individuals with MDD. All samples consisted of medication-free individuals, except for one study that did not exclude participants based on medication status (Narayan et al., 2022). Some studies used a minimal washout period of 2 or 3 weeks (Brennan et al., 2017, Godlewska et al., 2015, Sanacora et al., 2002, Taylor et al., 2012a), whereas another study used a longer washout period of 8 weeks (Block et al., 2009). One study only included participants with MDD who had not received medication for one year (Smith et al., 2021) and one study included only participants who were treatment-naive or had minimal exposure (<2 weeks lifelong) to antidepressant treatment (Draganov et al., 2020). The pgACC was assessed 4 times, the dorsal anterior cingulate cortex (dACC) twice, and the OCC also twice (Table 1, Fig. 2b).

##### 3.1.2.1 Pregenual anterior cingulate cortex

Taylor et al. (2012a) found no differences between subchronic 7-day treatment with escitalopram (SSRI; 10 mg/day; n = 21) vs. placebo (n = 19) in Glx concentrations. Brennan et al. (2017) investigated the effect of 6 weeks (chronic) open-label citalopram (SSRI; 20–40 mg/day) treatment in 16 participants with MDD on Glu, Gln, Gln/Glu and GABA concentrations at multiple time points after treatment onset (day 3, 7, and 42). No differences compared to baseline were observed for Glu, Gln or Gln/Glu for any of these time points. However, they found a decrease in GABA from baseline to day 3 of citalopram treatment. One of the few randomized, placebo-controlled studies (n = 39 SSRI; n = 41 placebo) reported no pre-post differences in Glx or GABA following chronic 8-week treatment with escitalopram (SSRI; 10–30 mg/day) or placebo (Narayan et al., 2022). As a marker for excitatory-inhibitory balance, the Glx/GABA ratio was also investigated, but no changes therein were observed. In a one-year follow-up, Draganov et al. (2020) compared Glu, Glx and GABA levels before and after one year of chronic treatment (variable, mostly SSRI; ∼10 mg/day) in 18 participants with MDD, but also found no significant change over time for these metabolites.

##### 3.1.2.2 Dorsal anterior cingulate cortex

An observational study assessed Glu levels in 14 individuals with MDD before and after subchronic 4 weeks of treatment (variable; SSRI, SNRI, TCA, atypical neuroleptics, or a combination) (Grimm et al., 2012). They did not observe a time-dependent change in Glu levels. Smith et al. (2021) assessed the effects of chronic 10–12 weeks citalopram (SSRI; 10–40 mg/day) treatment on Glu and GABA in 9 participants with late-life depression at 7 T. They likewise did not observe any differences between baseline and post-treatment measurements.

##### 3.1.2.3 Occipital cortex

One study found increased occipital GABA levels after at least 5 weeks of chronic citalopram (SSRI; ∼24 mg/day; n = 3) or fluoxetine (SSRI; ∼27 mg/day; n = 8) treatment compared to baseline in participants with MDD (Sanacora et al., 2002). In contrast, Godlewska et al. (2015) did not find altered concentrations of Glu, Gln or GABA in 39 participants with MDD after chronic 6-week citalopram treatment (SSRI; 10 mg/day; pre-post comparison).

##### 3.1.2.4 Other brain regions

In the posterior cingulate cortex (PCC), Block et al. (2009) found no group differences (pre) nor treatment effects (post) following 8-weeks chronic citalopram (SSRI; ∼20 mg/day; n = 5) or nortriptyline (TCA; n = 5) on hippocampal Glx and Gln. Another study in 9 individuals with late-life depression also did not observe pre-post differences in PCC Glu or GABA following chronic 10–12 weeks citalopram (SSRI; 10–40 mg/day) treatment (Smith et al., 2021). In the dorsolateral PFC (dlPFC), no changes in response to subchronic 4-week treatment were observed for Glu in 14 individuals with MDD (Grimm et al., 2012).

#### 3.1.3 Relationship between metabolite measures and symptoms

In the pgACC, Brennan et al. (2017) observed a significant positive association between clinical improvement from baseline to day 42 and change in GABA levels from baseline to day 3, and from baseline to day 7 in 16 participants with MDD. Additionally, lower baseline GABA levels were associated with greater clinical improvement. In contrast, another study found no correlation between Glx, GABA, or Glx/GABA and clinical symptoms, adjusted for treatment type (placebo vs. SSRI), in 81 participants with MDD (Narayan et al., 2022). Similarly, a one-year follow-up study did not find significant relationships between changes in symptom severity and Glx, Glu, or GABA levels in 18 individuals with MDD (Draganov et al., 2020). In the hippocampus, Block et al. (2009) observed no correlation between baseline Glx or Gln with baseline symptom severity scores in 10 participants with MDD. In the occipital cortex, Sanacora et al. (2002) reported no correlation between change in occipital GABA and change in symptom severity scores, nor a correlation between pre-treatment GABA levels and change in symptom severity in 11 individuals with MDD at 2.1 T. Similarly, another study reported no correlation between change in symptom severity and change in occipital Glu, Gln, or GABA level in 39 participants with MDD (Godlewska et al., 2015). Likewise, Grimm et al. (2012) found no association in 14 participants with MDD between Glu in the dACC or dlPFC and symptom severity at baseline or after treatment. Finally, Smith et al. (2021) found that decreases in PCC Glu, measured at 7 T, were associated with improvement in depressive symptoms in their modest sample of 9 individuals with MDD.

### 3.2 (Es)ketamine

Fourteen studies investigated the effect of (es)ketamine administration on glutamatergic and GABAergic metabolite concentrations (Table 2, Fig. 3). Results on other metabolites are included in the Supplementary Results and Table S3.

#### 3.2.1 (Es)ketamine in healthy volunteers

Twelve studies recruited healthy volunteers. Of these studies, six investigated metabolite levels in the pgACC, five in the dACC, two in the PCC, and two in the hippocampus. These are reported separately (Table 2, Fig. 3a). Studies varied with respect to the type of ketamine used, with 7 studies administering racemic ketamine (racemic mixture of the R- and S-enantiomer of ketamine), 2 studies administering esketamine (the S-enantiomer of ketamine), and 3 studies not specifying the type of ketamine used. Additionally, the infusion regimens were heterogeneous across studies, with some studies employing a single infusion and some separating the dosing regimen into bolus and infusion. Doses reported in the text reflect the (estimated) total dose.

##### 3.2.1.1 Pregenual anterior cingulate cortex

A placebo-controlled study investigating the effect of intravenous racemic ketamine (0.5 mg/kg) on Glx, Glu and Glx/Glu did not report any significant difference between ketamine (n = 8) and placebo (n = 9) groups, nor a time effect of ketamine in the 40 min after infusion (Taylor et al., 2012b). Similarly, a study investigating Glx and GABA concentrations found no significant pre-post changes in Glx, GABA or GABA/Glx 2 h post-infusion of racemic ketamine (0.8 mg/kg; n = 25) (Silberbauer et al., 2020). In contrast, Javitt et al. (2018) found an increase in Glx concentration in the pgACC in the ketamine (n = 31) compared to placebo (n = 16) group, but only in the first 15 min of a 60-minute racemic ketamine infusion (0.66 mg/kg). Notably, the change from baseline in Glx over the entire infusion period was not significantly different between treatment groups. Gärtner et al. (2022) reported a trend increase in Glu, but no changes in Gln or Gln/Glu 25 min after esketamine infusion compared to a baseline session (∼0.4 mg/kg) in 17 participants.

The only 7 T study that assessed neurometabolism 1 h post-infusion of racemic ketamine (0.5 mg/kg) found no change in Glu, Gln or Gln/Glu in the pgACC for both placebo (n = 14) and ketamine (n = 12) groups (Li et al., 2017). Interestingly, at 24 h post-infusion a significantly increased Gln/Glu ratio (but not Glu or Gln separately) was observed compared to baseline, which was significantly larger in the ketamine group. In contrast, a randomized, placebo-controlled crossover 7 T study found no differences between racemic ketamine (0.5 mg/kg; n = 17) and placebo infusions for Glu, Gln, and Gln/Glu 24 h after ketamine administration (Evans et al., 2018). In contrast, another placebo-controlled 7 T study observed higher Glu in the racemic ketamine (0.5 mg/kg; n = 40) group compared to the placebo group (n = 40) 24 h after administration (Colic et al., 2019).

##### 3.2.1.2 Dorsal anterior cingulate cortex

Li et al. (2017) assessed Glu, Gln and Gln/Glu at 7 T at 1 h and 24 h post-infusion in the dACC, but found no significant differences between ketamine and placebo groups. Rowland et al. (2005) reported an increase in Gln, but not Glu, measured at 4 T, from baseline to during bolus infusion of ketamine (up to ∼ 0.5 mg/kg; n = 10); although this increase was not reported during maintenance infusion. In contrast, Stone et al. (2012) showed an increase in Glu, but not Gln or Glx after ketamine infusion (∼0.7 mg/kg; n = 13; pre-post comparison). Another study did not find any pre-post effects of esketamine (0.29 mg/kg) on Glu, Gln and Glx, neither after the bolus nor in the maintenance phase in 25 participants (Bojesen et al., 2018). Moreover, the only study using MRSI reported no ketamine-induced alterations (pre-post) in Glx, GABA or GABA/Glx (Silberbauer et al., 2020).

##### 3.2.1.3 Posterior cingulate cortex

Bednarik et al. (2021) measured PCC Glu, Gln, Glx and Glu/Gln levels of 12 participants before and 3 h after a single racemic ketamine (0.5 mg/kg) infusion. No differences were observed between baseline and post-infusion measurements. The 3D-MRSI study of Silberbauer et al. (2020) also found no pre-post effects of ketamine administration on Glx, GABA, or GABA/Glx.

##### 3.2.1.4 Hippocampus

Kraguljac et al. (2017) investigated the effect of racemic ketamine (∼0.5 mg/kg; n = 15) on hippocampal Glx measured during infusion. Compared to baseline, Glx levels during infusion were increased in the ketamine, but not in the placebo group. Silberbauer et al. (2020) reported a pre-post ketamine reduction in GABA.

##### 3.2.1.5 Other brain regions

GABA levels in the thalamus were not altered following ketamine infusion compared to baseline (Stone et al., 2012). Finally, Silberbauer et al. (2020) did not find pre-post effects of ketamine on the thalamus, insula or putamen.

#### 3.2.2 (Es)ketamine in individuals with MDD

Only 3 of the included studies, assessed the effect of ketamine administration on metabolite levels in participants with MDD. All studies included medication-free individuals with MDD (drug-free for at least 2 weeks) and administered a single infusion of 0.5 mg/kg racemic ketamine (Table 2, Fig. 3b).

##### 3.2.2.1 Pregenual anterior cingulate cortex

Milak et al. (2016) observed an increase in Glu during, but not immediately after, a 40-minute infusion compared to baseline in 11 participants with MDD. They also reported increased GABA levels only 13 and 26 min after the start of the infusion. A 7 T study by Evans et al. (2018) in 20 participants with MDD observed a trend increase in Glu 24 h after a ketamine infusion, but no changes in Gln or Glu/Gln.

##### 3.2.2.2 Occipital cortex

Valentine et al. (2011) assessed the effect of an *i.v.* infusion of racemic ketamine on Glu, Gln and GABA 3 h and 48 h post-infusion, measured at 4 T. They did not observe an effect of ketamine compared to placebo on any metabolite at either time point.

#### 3.2.3 Relationship between (es)ketamine administration or treatment and symptoms

The 4 T study by Valentine et al. (2011) in 10 participants with MDD indicated that changes in occipital Glu, GABA, and Gln were not significantly correlated with changes in depressive symptoms after ketamine administration. This was observed for metabolite concentrations both 3 h and 48 h post-infusion. Additionally, baseline measures of glutamate, GABA and glutamine were not correlated with symptom severity scores at any time point. In the pgACC, Milak et al. (2016) reported that neither Glx nor GABA changes correlated with clinical response to ketamine.

### 3.3 Risk of bias assessment of included studies

Twelve studies were regarded as both controlled intervention and before-after studies with no control group. Both quality assessment tools were completed for these studies.

The NIH Quality assessment tool *for controlled intervention studies* was conducted for 14 studies. Results from this assessment tool are visualized in Table S4 (Supplementary Results). Of the SSRI/SNRI studies, 2 studies were rated as high quality (i.e., low risk of bias), and 5 as fair quality (i.e., fair risk or bias). For the (es)ketamine studies, 4 were rated as high quality, 2 as fair quality, and one study as poor quality (i.e., high risk of bias).

For 27 studies, the NIH Quality assessment tool *for before-after studies with no control group* was used (Table S5, Supplementary Results). All 13 SSRI/SNRI studies were rated as high quality, whereas for (es)ketamine, 11 studies were rated as high quality and three as fair quality.

The MRS-Q tool was used to assess the quality of the ^1^H-MRS design. Outcomes of this tool are visualized in Table S6 (Supplementary Results). For most studies only minor deviations from the quality criteria in the MRS-Q were observed. However, some studies showed major deviations which may impede the reliability of metabolite concentration estimates for these studies, which was mostly applicable to two studies measuring GABA concentrations at 3 T using non-edited sequences.

## 4 Discussion

In this systematic review, we investigated changes in glutamatergic and GABAergic metabolite levels, measured using ^1^H-MRS, in response to SSRIs, SNRIs, or (es)ketamine. Studies investigating administration of, or treatment with SSRIs or SNRIs were generally underpowered and yielded widely varying results, with no consistent findings across voxel locations, populations, or investigated metabolites. For (es)ketamine, results are marginally more consistent, suggesting that (es)ketamine increases glutamate levels in the pgACC and dACC at study-specific, but inconsistent, time points after administration. However, as the majority of included studies reported no effect of medication on metabolite levels, the observed trend should be interpreted with caution. The SSRI/SNRI studies exhibited comparable variability in the association between metabolite levels and clinical outcomes, without clear emerging trends. No evident association with clinical outcomes was observed in the (es)ketamine studies.

### 4.1 Metabolite changes in response to SSRIs, SNRIs, and (es)ketamine across brain regions

SSRIs, SNRIs, and (es)ketamine have different neurotransmitter receptor targets that are differentially expressed throughout the brain, and may exert therapeutic effects through different pathways. As such, the brain region studied might be an important contributor to the effects of these antidepressants on glutamatergic and GABAergic metabolites. Whereas some of the investigated brain regions may be directly affected by antidepressant medication, changes in other regions (e.g., the OCC) might be the result of medication-induced changes elsewhere. The pgACC is considered to be a critical brain region involved in the pathophysiology of MDD (Godlewska et al., 2018, Pizzagalli et al., 2018) and TRD (Salvadore et al., 2009, Salvadore and Zarate, 2010). As part of the limbic network, it plays a significant role in emotional processing (Pizzagalli, 2011). In individuals with MDD, the pgACC is thought to exhibit hyperactivity (Mayberg et al., 2005, Mayberg et al., 1999, Seminowicz et al., 2004), which is reduced after treatment with SSRIs (Drevets et al., 2002, Mayberg et al., 2000). Notably, both SSRIs (Arnone et al., 2018) and (es)ketamine (Gärtner et al., 2022) increase connectivity between the pgACC and dorsolateral prefrontal cortex. Likewise, (es)ketamine increases BOLD and cerebral blood flow, and alters connectivity in the dACC (Bryant et al., 2019, De Simoni et al., 2013, Gärtner et al., 2022).

Therefore, the pgACC may be a key region in the shared biological pathway targeted by different types of antidepressant medication. Indeed, computational modeling suggests that glutamatergic disturbances in the pgACC (i.e., slower glutamate clearance) underlie systems-level alterations in the PFC in MDD (Ramirez-Mahaluf et al., 2017). They additionally show that SSRIs, through serotonin (5-HT) 1A receptor-mediated hyperpolarization, could normalize these disturbances. (Es)ketamine-related increases in PFC glutamate are supported by preclinical studies showing increased extracellular glutamate in rodents (Chowdhury et al., 2012) and results of a ^13^C-MRS study, which showed that (es)ketamine increases glutamate-glutamine cycling in the medial PFC (Abdallah et al., 2018). Although this offers a potential shared neurobiological pathway through which SSRIs, SNRIs, and (es)ketamine could exert their therapeutic effects, the ^1^H-MRS results of our systematic search do not provide support for this.

For SSRIs and SNRIs, none of the studied brain regions yielded consistent results, suggesting that a) effects of these antidepressants do not affect the glutamate/GABA systems, b) effects on glutamate/GABA systems cannot readily be determined with ^1^H-MRS, c) effects are elicited in different brain regions, or d) the described studies lack statistical power to detect the effects of these antidepressants on metabolite levels. Moreover, the large heterogeneity observed in study design (e.g., medication dose, route of administration, and treatment duration) complicates interpretation of ^1^H-MRS findings following SSRI/SNRI administration. The (es)ketamine studies showed less variation in in study design than studies investigating SSRIs or SNRIs. For these studies, a somewhat more consistent pattern emerges where findings show a tentative increase in glutamatergic neurometabolite levels in several subregions of the ACC, while fewer effects were observed in other brain regions such as the PCC and OCC.

From a methodological standpoint, the quality of data obtained from different voxel locations may not be uniform. For instance, the OCC is not typically investigated in MDD but it can provide high-quality spectra with relative ease. Conversely, while the hippocampus and subgenual ACC (sgACC) are widely regarded as important brain regions in the pathophysiology of MDD, they are notoriously difficult to shim and thus acquire spectra with acceptable linewidths from. In addition, ^1^H-MRS voxels are relatively large, thereby frequently including several smaller anatomical subregions. This is especially problematic in e.g. the ACC, where different subregions have been shown to exhibit distinct expression patterns of glutamatergic receptor subtypes (Dou et al., 2013, Palomero-Gallagher et al., 2009). These methodological limitations must be taken into account when interpreting ^1^H-MRS studies.

Additionally, it is important to acknowledge and address the potential sources of changes in metabolite concentrations following antidepressants. Metabolite concentrations detected by ^1^H-MRS are reflective of Glu, Gln, and GABA present in synaptic, extracellular, and astrocytic pools, which currently cannot be distinguished from each other. Several hypotheses have been proposed to explain the dynamic changes in Glu and GABA, including alterations in metabolic turnover and shifts between different compartments. One hypothesis posits that there is an augmentation of neurometabolites due to changes in their synthesis (Mangia et al., 2012, Mangia et al., 2009). Conversely, the second hypothesis suggests that metabolites might relocate from presynaptic vesicles, where they remain undetectable by ^1^H-MRS, to extracellular and cytosolic pools, where they become accessible for detection by ^1^H-MRS (Lea-Carnall et al., 2023). However, to what extent these dynamic changes extrapolate to (long-term) drug-induced changes in metabolite concentrations is not yet fully understood.

### 4.2 Experimental design considerations across studies

For SSRIs and SNRIs, it is generally accepted that a treatment duration of several weeks is necessary to exert therapeutic effects. We therefore investigated differences between acute, subchronic, or chronic SSRI/SNRI administrations. Differences in glutamatergic or GABAergic neurometabolism with varying treatment durations could be driven by adaptive processes at the 5-HT synapse in response to prolonged treatment (Sangkuhl et al., 2009), or through changes in brain-derived neurotrophic factor-driven neural plasticity (Björkholm and Monteggia, 2016). However, we showed no evidence for an effect of treatment duration of SSRIs or SNRIs on glutamate/GABA levels. It should however be noted that most studies administering SSRIs and SNRIs were conducted in individuals with MDD, as part of treatment studies. Only one study investigated acute effects of SSRIs on glutamatergic or GABAergic metabolites, which impedes a full exploration of the effect of treatment duration in this review.

For (es)ketamine, the effects of its administration on the brain over time are not as well understood. Although improvement in clinical symptoms occurs rapidly following (es)ketamine infusion and is thought to sustain for several days, preclinical studies have shown both rapid and transient increases in glutamate release (a glutamate “burst”) following (es)ketamine administration in the rat PFC (Moghaddam et al., 1997) and in humans (Homayoun and Moghaddam, 2007). Likewise, our findings show an apparently inconsistent effect of (es)ketamine infusion over time on glutamate levels, with both significant effects and null findings observed shortly after (es)ketamine infusion, as well as 24 h later. Those studies conducting multiple post-infusion measurements report changes at specific timepoints, which are frequently not sustained when averaging over the entire post-infusion period. However, as all (es)ketamine studies used a single intravenous administration, predominantly in healthy volunteers, we were not able to determine long-term effects of (sub)chronic (es)ketamine administration. Crucially, a full mechanistic explanation of the direct and indirect impact of (es)ketamine on the human brain is lacking. Besides NMDA antagonism, (es)ketamine has been shown to additionally affect multiple neurotransmitter systems, including the serotonergic (Gigliucci et al., 2013), noradrenergic (Tso et al., 2004), dopaminergic (Kokkinou et al., 2017), cholinergic (Moaddel et al., 2014) and opioid (Klein et al., 2020) systems. The interplay between these systems and (possibly subsequent) alterations in glutamate and GABA levels remains to be resolved.

### 4.3 Differences across diagnostic groups in metabolite changes

Although previous studies have shown differences in glutamatergic and GABAergic metabolites in participants with MDD compared to healthy volunteers (Lener et al., 2017, Moriguchi et al., 2019, Ritter et al., 2022, Yüksel and Öngür, 2010), we observe no effect of diagnostic group on the response to SSRIs, SNRIs and (es)ketamine. However, it is important to note that there is little overlap of investigated brain regions between these two groups. We also cannot draw conclusions on how differences in in- and exclusion criteria affected the impact of antidepressants on neurometabolism, due to the small number of studies and variety in criteria. Nevertheless, it would be interesting for future studies to e.g. assess differences between patients with specific comorbidities, or those within specific subgroups of MDD, as esketamine is approved specifically for treatment of TRD.

### 4.4 Association of metabolite levels with clinical response

Numerous ACC subregions are implicated in terms of predicting antidepressant treatment response. For example, studies have shown that response is predicted by the sgACC baseline metabolism (Nugent et al., 2014), pgACC gray matter volume (Chen et al., 2007), and pgACC reactivity (Strege et al., 2023) and is correlated with connectivity between the pgACC and dACC (Kozel et al., 2011).

For SSRIs and SNRIs, the findings show mixed results regarding the correlation between changes in Glu and GABA levels and clinical improvement in patients treated with SSRIs and SNRIs. Some, but not all studies found that increases in pgACC GABA levels were associated with clinical improvement. One study found that decreases in PCC Glu were associated with a reduction in depressive symptoms. However, in the OCC and dACC/dlPFC, no such association was evident. Our results furthermore suggest that changes in Glu, GABA, and Gln concentrations are not significantly correlated with changes in depressive symptoms after (es)ketamine administration, regardless of voxel placement. However, most studies were conducted in small samples with considerable variation in clinical tools used to determine symptom severity. Additionally, it remains difficult to reliably determine treatment efficacy in these cohorts, particularly when employing treatment periods shorter than 6 weeks.

### 4.5 Study quality

Half of the studies on SSRIs and SNRIs used randomized, placebo-controlled designs, while the other half used an open-label design. Despite preference for a randomized placebo-controlled design, no differences were observed between study designs. For (es)ketamine studies, most used a placebo-controlled design, but conducting a blinded placebo-controlled study for (es)ketamine can be challenging due to its potent subjective effects (Ingram et al., 2018). The quality of evidence from the included studies was generally fair to good, with many being of moderate risk of bias due to issues such as inadequate blinding or incomplete outcome data. Additionally, many of the included studies had small to moderate sample sizes and the majority did not show power calculations. Crucially, previous power analyses showed that a sample size of 18 was necessary to detect a 10 % difference in metabolite levels in a crossover design (Hansen et al., 2016). To detect a 15 % change in GABA in the ACC, a sample size of 27 would be necessary using a between-group design and 36 for a within-subject cross-over design (Sanaei Nezhad et al., 2019). Although these estimates cannot readily be compared to other studies, we present these findings here to illustrate that the majority of included studies had sample sizes well below these estimates and might therefore be underpowered.

Considering ^1^H-MRS methodology, the included studies did not reveal any clear effect regarding the MRS sequence used. However, we found moderate evidence that studies utilizing higher field strengths (7 T) were more likely to detect an increase in Glu after (es)ketamine administration in the pgACC. In contrast, studies that used lower field strengths (≤ 3 T) seem more likely to report no changes or only trends. Moreover, substantial differences in (reporting of) ^1^H-MRS acquisition, processing and analysis method existed between studies, which complicates the estimation of the quality and risk of bias of the included studies. Variation in reporting was most evident for the reference method used, the use of in-house analysis tools and limitations in reporting of software and processing steps used for data analysis. This highlights the importance to scan at higher field strengths, standardize acquisition protocols and increase transparency about analysis strategies to ensure the reliability and reproducibility of results.

Some of the included studies deviated to varying extent from the quality criteria stated in the MRS-Q. Two studies assessing GABA concentrations at 3 T used unedited sequences, although such sequences usually do not provide sufficient spectral resolution to resolve the overlap between the main GABA peaks and peaks from other neurometabolites (Mullins et al., 2014). To resolve this issue, spectral editing techniques are currently regarded as most powerful and reliable tool for the quantification of GABA levels. However, it should be noted that some discussion still remains on the quantification reliability of GABA levels with varying (non)-edited sequences and field strengths (for a review, see Puts and Edden, 2012).

### 4.6 Future outlook

Several important areas to study the effects of antidepressants on the glutamate/GABA systems using ^1^H-MRS need to be considered. First, establishing standardized study and acquisition protocols and increasing transparency regarding analysis strategies is crucial to promote reliability and reproducibility. Recently, many efforts have been made in terms of standardization and transparency of ^1^H-MRS research. We have provided an overview of current experts’ consensus recommendations resulting from these efforts, combined with additional methodological considerations for pharmacological ^1^H-MRS studies, in Table 3. Additionally, measuring metabolites dynamically over time could provide valuable insights into the temporal dynamics of these systems (Lea-Carnall et al., 2023, Mullins et al., 2005, Stanley and Raz, 2018), particularly as changes in metabolites in response to (es)ketamine administration might occur at specific time points only (but not across the entire ^1^H-MRS acquisition period). A final promising avenue is to combine MR spectroscopy with whole brain functional imaging techniques, such as functional MRI. This could provide a more complete picture of the relationship between metabolic activity and brain function (Bednařík et al., 2015, Betina Ip et al., 2019, Koush et al., 2022).

**Table 3:** Methodological considerations for future studies.

**Study design**	• Include a valid and realistic power calculation to estimate the sample size • Collect multiple time points before and after the administration of, or treatment with, medication • Include a placebo condition/session in favour of a pre-post design	
**Acquisition**	• Use appropriate sequences for the available magnetic field strength to quantify the metabolite of interest: ○ GABA: edited MRS at 3T, e.g. MEGA-PRESS (Mullins et al., 2014; Peek et al., 2020) ○ Glutamate: e.g. PRESS or sLASER at 3T (Glx), and STEAM or sLASER (Glu) at 7T (Öz et al., 2021, Peek et al., 2020) • Benchmark the 1H-MRS protocol to existing quality guidelines in terms of SNR, linewidth and CRLBs (Juchem and de Graaf, 2017) • Export individual transients instead of the averaged spectrum to allow optimal processing and temporal analysis of the data	
**Analysis**	*Processing* • Follow processing recommendations, regarding e.g. eddy current correction, motion correction, frequency and phase drift correction, alignment/subtraction of sub-spectra, and nuisance peak removal (Near et al., 2021) *Fitting and analysis* • Follow fitting and analysis steps based on experts’ consensus (Near et al., 2021) • Employ dynamic fitting approaches for i.v. (Clarke et al., 2022; Tal, 2023)	
**Reporting**	*Study design* • Report type of medication, timing of measurements, and total (estimated) dose	
	*Acquisition* • Report all relevant hardware • Fully report acquisition parameters *Analysis* • Describe the analysis method in detail; specifically any deviations from experts’ consensus • Provide quality measures, including sample spectrum	In accordance with MRSinMRS (Lin et al., 2021)
**Open Access**	• Preregister the analysis • Share datasets • Share basis sets • Share analysis code	On e.g MRSHub or GitHub

Abbreviations: CRLB: Cramér-Rao lower bound; GABA: γ-aminobutyric acid; MRSinMRS: Minimum Reporting Standards in MRS; SNR: signal-to-noise ratio.

### 4.7 Conclusion

Our systematic collection and review of ^1^H-MRS findings did not elucidate a clear effect of treatment with or administration of SSRI or SNRIs on GABAergic or glutamatergic metabolite levels in both healthy volunteers and individuals with MDD. Our findings suggest (es)ketamine-induced alterations in glutamatergic metabolites in ACC regions in both participant groups, but the timing of these effects remains to be resolved. Unfortunately, the many null findings due to generally underpowered studies complicate a reliable interpretation of these emerging trends. We did not find evidence for a comparable or shared effect of SSRIs and SNRIs, and (es)ketamine on glutamatergic and GABAergic neurometabolism. The observed inconsistencies might (also) be related to differences in study design, the studied brain region, and ^1^H-MRS acquisition protocols and analysis approaches. Together, this highlights the need to standardize these aspects in future research. Additionally, as the response to these types of medication might be dynamic in nature, studies using functional ^1^H-MRS designs to assess both transient and sustained changes might elucidate the direct and downstream effects of these mechanistically distinct types of antidepressants. Finally, studies combining ^1^H-MRS with other imaging modalities, such as fMRI, could pave the way to understand the relation between changes in neurotransmission induced by these types of medication and its effect on the functional response of the brain.

## Author contributions

DB and AS wrote the pre-registered protocol on Prospero. ER and LR contributed to the drafting of the final version of this protocol. DB conducted the systematic search, DB and AS conducted the screening of articles, data extraction and quality screening. DB wrote the first draft of the manuscript. All authors contributed to the interpretation of findings and drafting the manuscript. All authors approved the final version of the manuscript.

## Funding

AS is supported by an NWO ZonMw Veni 016.196.153.

## Declaration of Competing Interest

The authors declare that they have no known competing financial interests or personal relationships that could have appeared to influence the work reported in this paper.

## Data Availability

No data was used for the research described in the article.
